# Molecular understanding of unusual HbE-β^+^-thalassemia with Hb phenotype similar to HbE heterozygote: simple and rapid differentiation using HbE levels

**DOI:** 10.1080/07853890.2023.2267054

**Published:** 2023-10-10

**Authors:** Wittaya Jomoui, Surada Satthakarn, Sitthichai Panyasai

**Affiliations:** a Department of Pathology, Maha Chakri Sirindhorn Medical Center, Srinakharinwirot University, Ongkharak, Nakhon Nayok, Thailand; b Clinical Research Centre, Srinakharinwirot University, Nakhon Nayok, Thailand; c Faculty of Allied Health Sciences, Burapha University, Chonburi, Thailand; d Department of Medical Technology, School of Allied Health Sciences, University of Phayao, Phayao, Thailand

**Keywords:** Hemoglobin E, Hemoglobin F, Heterozygous HbE, HbE-β^+^-thalassemia, Diseases, *Xmn*I polymorphism

## Abstract

**Background:**

Low HbF expression in HbE-β^+^-thalassemia may lead to misdiagnosis of HbE heterozygosity. We aimed to characterize the β*-* and α-globin genes and the modifying factors related to HbF expression in patients with an Hb phenotype similar to that of HbE heterozygotes. Furthermore, screening tools for differentiating HbE-β^+^-thalassemia from HbE heterozygotes have been investigated.

**Participants and Methods:**

A total of 2133 participants with HbE and HbA with varying HbF levels were recruited. Polymerase chain reaction-based DNA analysis and sequencing were performed to characterize β- and α-globin genes. DNA polymorphism at position −158 nt 5′ to ^G^γ-globin was performed by *Xmn*I restriction digestion. Receiver operating characteristic (ROC) curves were constructed using the area under the curve (AUC). Cutoff values of HbA_2_, HbE, and HbF levels for the differentiation of HbE-β^+^-thalassemia from HbE heterozygotes were determined.

**Results:**

Five β^+^-thalassemia mutations *trans* to β^E^-gene (β^−87(C>A)^, β^−31(A>G)^, β^−28(A>G)^, β^19(A>G)^, and β^126(T>G)^) were identified in 79 patients. Among these, 54 presented with low HbF levels, and 25 presented with high HbF levels. ROC curve analysis revealed an excellent AUC of 1.000 (95% confidence interval:1.000–1.000) for HbE levels, and a cut-off point of ≥35.0% had 100.0% sensitivity, specificity, and Youden’s index for differentiating HbE-β^+^-thalassemia from HbE heterozygotes. The proportion of α-thalassemia mutations was 46.3 and 8.0% among HbE-β^+^-thalassemia patients with low and high HbF levels, respectively. Two rare α-thalassemia mutations (Cap +14(C>G) and initiation codon (ATG>-TG)) of α_2_-globin genes were identified. The genotype and allele of the polymorphism at −158 nt 5′ to ^G^γ-globin was found to be negatively associated with HbF expression.

**Conclusions:**

HbE-β^+^-thalassemia cannot be disregarded until appropriate DNA analysis is performed, and the detection of α-thalassemia mutations should always be performed under these conditions. An HbE level ≥35.0% may indicate screening of samples for DNA analysis for HbE-β^+^-thalassemia diagnosis.

## KEY MESSAGES

1. HbE-β^+^-thalassemia displays a wide range of HbF expression, which may lead to the misdiagnosis of HbE heterozygosity in patients whose Hb analysis shows HbE and HbA. α-Thalassemia may be a major factor associated with decreased secondary activation of HbF expression in the disease.
2. HbE may be a potential indicator for effectively differentiating HbE-β^+^-thalassemia from HbE heterozygotes.
3. The high proportion and heterogeneity of α-thalassemia mutations found in patients with HbE-β^+^-thalassemia evoke a complex thalassemia syndrome, requiring complete DNA analysis.

## Introduction

β-Thalassemia is a group of genetic disorders characterized by a quantitative deficiency of β-globin chain production and is further classified as either β^0^- or β^+^-thalassemia, based on the absence or reduction of β-globin chain production, respectively [1,2]. Hemoglobin E (Hb) E is the most common hemoglobinopathy in Southeast Asia and is caused by a point mutation in codon 26 (GAG to AAG) of the β-globin gene, which results in the substitution of lysine for glutamic acid. This mutation creates abnormal splicing within exon 1 of the β-globin gene, resulting in reduced production of β-globin, which manifests in the clinical phenotype of a mild form of β-thalassemia, β^+^-thalassemia [3]. The clinical manifestation of HbE is dependent on co-inheritance of the β-globin gene defect. Heterozygous HbE is asymptomatic with normal or slight microcytosis, while homozygous HbE is clinically normal with remarkable microcytosis of red blood cells (RBCs), similar to that observed in β-thalassemia carriers. The interaction of HbE with β-thalassemia can cause HbE-β-thalassemia disease, the most common β-thalassemic disease worldwide, with the highest frequencies observed in Asia and throughout Southeast Asia, particularly in Thailand, where it is common for individuals to inherit alleles for both hemoglobin E (HbE) and beta-thalassemia [4,5]. HbE-β-thalassemia produces a clinical phenotype ranging from mild to severe anemia, with Hb levels ranging from 2.5 to 13.3 g/dL [6]. HbE-β-thalassemia results from the co-inheritance of a β-thalassemia allele from one parent and the structural variant HbE from the other. The clinical phenotype of HbE-β^0^-thalassemia is usually more severe than HbE-β^+^-thalassemia. Hb levels of 9.4–11.1 g/dL have been observed in a cohort of Thai patients with pure HbE-β^+^-thalassemia [7]. A presumptive diagnosis of the two diseases can be made by Hb analysis: in HbE-β^0^ thalassemia, β^A^-globin chains are not synthesized, and the condition is characterized by the production of HbE and HbF without detectable HbA; HbE constitutes 30–70% of the hemoglobin with the remainder HbF. In HbE-β^+^-thalassemia, variable amounts of HbA are detected, in addition to HbE and HbF. Different β^+^ thalassemia mutations result in variable disease severity, reflecting different levels of HbA. Interestingly, in many cases of HbE-β^+^-thalassemia, HbF is 0.0–5.0% of the total Hb, making it undetectable or only mildly detectable [2]. Thus, HbE-β^+^-thalassemia with such phenotypes is difficult to differentiate from HbE heterozygotes by routine laboratory investigations based on hematological screening and Hb analysis, leading to misdiagnosis of the phenotype as HbE heterozygous, especially in areas where HbE and β-thalassemia are prevalent, such as Thailand. Misdiagnosis can lead to inaccurate genetic counseling and affect the prognosis of the disease. In such cases, appropriate DNA analysis is necessary for accurate diagnosis.

The molecular basis of HbE-β^+^-thalassemia with such a phenotype and modifying factors associated with decreased or abolished reactivation of the γ-globin gene leading to low HbF levels are not well understood. The genetic factors strongly associated with the regulation of HbF expression in β-thalassemia, sickle cell diseases, HbE-related disorders, and healthy individuals in various populations include three major loci: rs7482144 (^G^γ-*Xmn*I) polymorphism in the promoter region of ^G^γ-globin, rs4895441, rs9399137 (HBS1L-MYB), and rs4671393 (BCL11A) [8–10]. Understanding the molecular basis and genetic factors that modulate phenotypic expression is important for providing appropriate counseling and planning for prenatal diagnosis and patient management. Hence, a simple initial screening tool is warranted to identify appropriate samples from patients with presumed HbE heterozygosity by Hb analysis for further DNA testing for HbE-β^+^-thalassemia.

## Materials and methods

### Study participants and hematological analysis

**Figure 1.**
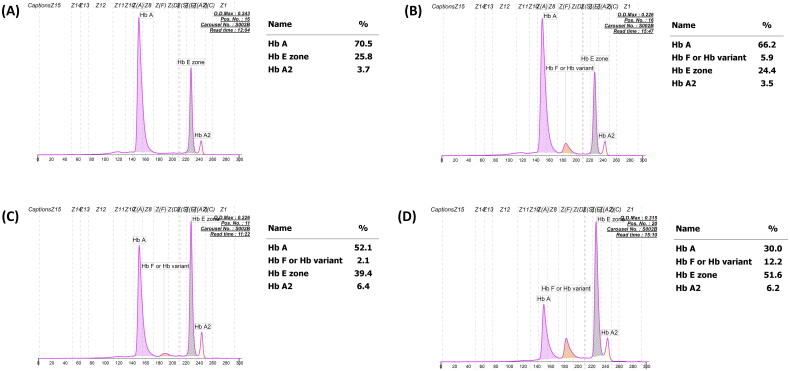
Representative capillary electropherograms of patients with classical heterozygous HbE with undetectable HbF (A), those with increasing HbF levels (B), HbE-β^+^-thalassemia with decreasing HbF expression (C), and classical HbE-β^+^-thalassemia with increasing HbF expression (D).

A total of 2133 unused ethylenediaminetetraacetic acid anticoagulated blood samples, whose Hb analysis showed HbE and HbA with varying HbF levels ranging from 0.0% to 40.0%, were selected from among the samples submitted for routine Hb analysis from October 2020 to September 2021 at the Division of Hematology, Lampang Hospital, Thailand (Figure 1). These included patients being evaluated for anemia or antenatal screening and those who had not received a transfusion for at least 3 months before blood collection. Hb analysis was performed using automated capillary electrophoresis (CE; Capillarys 2 Flex Piercing; Sebia SA, Lisses, France). Additionally, automated cation exchange high-performance liquid chromatography (HPLC) (VARIANT II system, Bio-Rad Laboratories, Hercules, CA, USA, using the β-Thalassemia Short Program) and HPLC (Premier Resolution system from Trinity Biotech, Bray, Country Wicklow, Ireland, using the high-resolution mode) were used to separate Hb from seven samples with β-chain variants. Hematological parameters of all samples were measured using an automated blood cell counter (Coulter T series; Beckman-Coulter Co., Fullerton, CA, USA) and were originally recorded at the hospital. A total of 1946 patients were assigned to be heterozygous HbE because Hb analysis revealed both HbE and HbA (Figure 1A), with an HbE level <30% being considered an acceptable range for heterozygous HbE [7,11–13]. The remaining 187 patients, comprising those whose Hb analysis showed the following outcomes:48 had HbF >5.0% and HbE < 30.0% (Figure 1B); 95 had HbF <5.0% and HbE > 30.0% (Figure 1C); and 44 had HbF >5.0% and HbE >30.0% (Figure 1D). These patients were selected for further analysis of β- and α-globin gene mutations.

The research protocol was approved by the Institutional Review Board (IRB) of the University of Phayao, Thailand (approval number:1.3/004/63), and all subjects were selectively recruited after obtaining informed consent. The study was performed in accordance with the relevant guidelines and regulations.

### Molecular characterization of the globin genes and genotyping of modifying factors related to HbF expression

Genomic DNA was prepared from peripheral blood leukocytes using the proteinase K method with a standard protocol [14]. β-thalassemia mutations were identified by direct DNA sequencing of the entire amplified β-globin gene, and the sequence was analyzed on an ABI PRISM™ 3130 XL analyzer (Applied Biosystems, Foster City, CA, USA), as described previously [15]. In addition, seven common α-thalassemia comprising α^0^-thalassemia (SEA, Thai, and Chiang Rai deletions), deletional α^+^-thalassemia (3.7 and 4.2 base kilobase [kb] deletions), Hb Constant Spring (HbCS; HBA2: c.427T>C), and Hb Paksé (HBA2: c.429A>T) mutations were also identified using appropriate polymerase chain reaction (PCR) methodology [16–19]. Samples with negative results for common α-thalassemia and the α_1_- and α_2_*-*globin gene mutations were characterized using PCR and direct DNA sequencing methods according to the manufacturer’s protocols, as described previously [20]. The inherent factors that influence HbF expression were determined by studying the −158 (C-T) ^G^γ *Xmn*I polymorphism (SNP rs7482144), a modifying factor strongly associated with increased HbF expression, using PCR-restriction fragment length polymorphism (PCR-RFLP) with *Xmn*I restriction enzyme [21].

### Identification of non-deletional α^+^-thalassemia mutations by the PCR-RFLP technique

The C to G substitution at the cap site nucleotide (nt) 14 [HBA2:c.-24C>G (or HBA1)] and the deletion of A in the translation initiation codon (ATG→-TG) [HBA2:c.1delA] in the α_2_-globin gene abolished the *Bsm*FI and *Fat*I restriction sites, respectively. These two mutations were subsequently confirmed using polymerase chain reaction-restriction fragment length polymorphism. Amplification of the 1085-base pair (bp) fragment specific for the α_2_-globin gene was performed using the specific primers C1 (5′-TGGAGGGTGGAGACGTCCTG-3′) and C3 (5′-CCATTGTTGGCACATTCCGG-3′) [22]. The amplified fragment was then digested to completion with *Bsm*FI (5′-GGGAC(N)_10_^▼^-3′) and *Fat*I (5′-^▼^CATG-3′) to identify the respective mutations. The restriction endonucleases *Bsm*FI and *Fat*I were purchased from New England Biolabs Inc. (Beverly, MA, USA). The digested fragments were analyzed using 2.0% agarose gel electrophoresis and visualized under ultraviolet light after ethidium bromide staining.

### Statistical analysis

Descriptive statistics including mean, median, and standard deviation were used to describe the overall characteristics of the participants. The proportion of modifying factors related to HbF expression was compared between the two groups using Fisher’s exact test. Receiver operating characteristic (ROC) curve analysis was performed to determine the optimal cut-off values of HbA_2_, HbE, and HbF. The diagnostic accuracy of each parameter was assessed using the area under the curve (AUC), sensitivity, specificity, positive and negative predictive values, and Youden’s index (YI) for each cut-off value. Statistical and ROC curve analyses were performed using SPSS software (version 22.0; Armonk, NY, IBM Corp). A *P*-value of less significance was set at *p* < 0.05.

## Results

**Figure 2.**
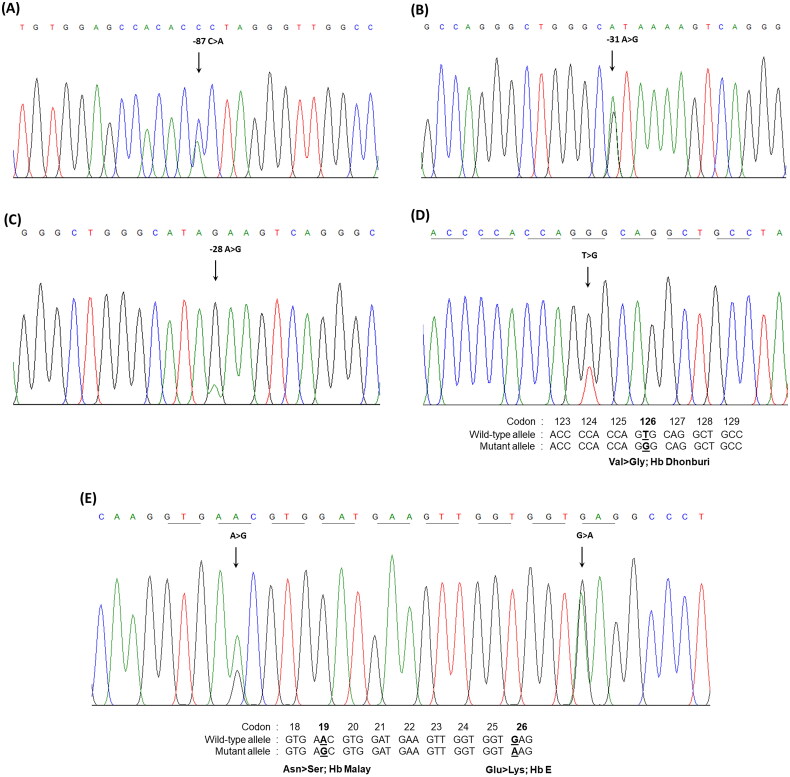
The molecular characterization of the β-globin gene performed by DNA sequencing. Mutations in the promotor region of β-globin gene; (A) nt-87(HBB:c.-137C>a), (B) nt-31(HBB:c.-81A>G), (C) nt-28(HBB:c.-78A>G), (D) Hb Malay (beta 19(B1) Asn>ser; HBB:c.59A>G), and (E) Hb Dhonburi (beta 126(H4) Val>gly;HBB:c.380T>G).

**Figure 3.**
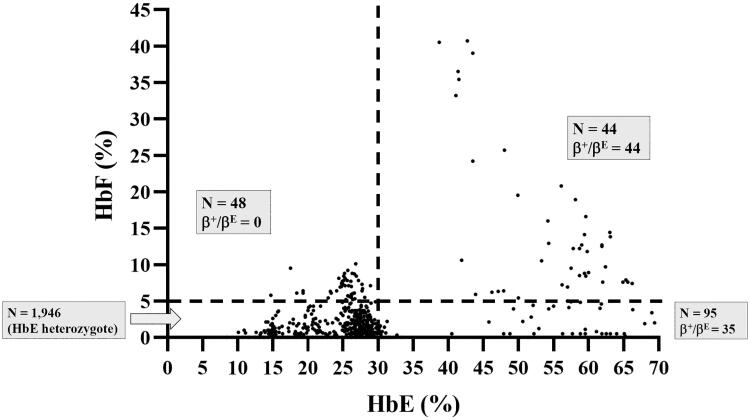
Distribution of HbF and HbE levels among the study population. The patients are Subdivided into four groups using the HbF and HbE levels obtained by capillary electrophoresis. Hb: hemoglobin.

The overall median age of the 2133 participants was 39 years (range: 1–98 years). Of these, 1946 patients were heterozygous for HbE, whereas the remaining 187 were completely diagnosed by DNA analysis. Molecular analysis of the β-globin mutations revealed that 79 patients (42.3%) carried β-thalassemia mutations identified *trans* to the β^E^ allele. These included three mutations in the promoter region of the β-globin gene, nt-87(HBB:c.-137C > A), nt-31(HBB:c.-81A > G), and nt-28 (HBB:c.-78A > G), and two missense mutations, Hb Malay (HBB:c.59A > G) and Hb Dhonburi (HBB:c.380T > G) (Figure 2). These mutations are responsible for β^+^-thalassemia [23]. The proportion of HbE-β^+^-thalassemia patients identified according to our selection criteria (using HbF and HbE levels) is shown in Figure 3. Among the 79 patients with HbE-β^+^-thalassemia, 35 (44.3%) had HbF levels less than 5.0% and HbE levels > 30.0%. The remaining 44 (55.7%) patients were identified in the group with HbF levels > 5.0% and HbE levels > 30.0% (Figure 3). These results indicate that the patients in our study with HbE-β^+^-thalassemia had a wide-range expression of HbF and HbE of 0.0–40.7% and 38.7–69.4%, respectively. Based on HbF levels, these patients were categorized into two groups: low HbF levels (<10.0%, *n* = 54) and high HbF levels (≥10.0%, *n* = 25), as shown in Table 1. Statistical analysis of hematological parameters and Hb analysis demonstrated that Hb, hematocrit (Hct), mean corpuscular volume (MCV), and mean corpuscular Hb (MCH) of patients with HbE-β^+^-thalassemia were significantly lower than those of patients with heterozygous HbE. Furthermore, significantly increased red cell distribution width (RDW) was observed in patients with HbE-β^+^-thalassemia. These hematological parameters were similar to those observed in patients with low and high HbF levels. Interestingly, we found one patient carrying a β-genotype with β^E^/β^−31(A>G)^ among the group with low HbF levels and secondary erythrocytosis (RBC count: 11.1 × 10^12^/L; Hb: 22.4 g/dL; Hct: 75.3%). This indicated the possibility of polycythemia vera (PV), as the values for the parameters were within the accepted range specified by the World Health Organization [24]. Extensive testing of *JAK2*V167F and *JAK2* exon 12 mutations, commonly found in PV, and the *CALR* gene mutation, rarely found in PV, was performed; however, these analyses were negative. Presumably, this patient had *JAK2* unmutated erythrocytosis. Therefore, comprehensive molecular analysis to identify probable specific gene mutations of *JAK2* unmutated erythrocytosis may provide a key for better diagnosis and explain the pathogenesis of the disease [25]. However, further analysis in the search for these specific gene mutations could not be performed in this study.

**Table 1.** Hematological parameters, HbE, HbA_2_, and HbF levels in HbE heterzygosity, HbE-β^+^-thalassemia with low HbF level, and HbE-β^+^-thalassemia with high HbF level. Data are expressed as means ± standard deviations (min-max).

Parameters	Heterozygous Hb E(*n* = 2054)	β^+^/Hb E with low Hb F (*n* = 54)	β^+^/Hb E with high Hb F (*n* = 25)	*p*-value
Age (years)	41.8 ± 24.1(<1 – 98)	44.5 ± 24.0(4 – 83)	39.8 ± 27.3(<1 – 78)	–
Male: Female	863:1191	26:28	16:9	–
RBC (10^12^/L)	4.5 ± 1.1(1.36 – 8.51)	5.3 ± 1.2*** (2.8 – 11.1)	4.7 ± 0.9(3.1 – 7.1)	<0.0001
Hb (g/dL)	10.6 ± 2.6(2.7 – 19.8)	9.8 ± 2.7* (5.4 – 22.4)	9.2 ± 1.5** (6.5 – 12.7)	0.0003
Hct (%)	32.5 + 7.8(9.5 – 59.3)	30.6 ± 8.7* (16.6 – 75.3)	29.2 + 4.6* (22.1 – 41.1)	0.0018
MCV (fL)	72.5 ± 9.1(43.7 – 120.9)	57.2 ± 6.7*** (45 – 89.4)	62.7 ± 7.8*** (51.2 – 81.0)	<0.0001
MCH (pg)	23.6 ± 3.5(11.3 – 40.6)	18.4 ± 2.1*** (14.0 – 29.0)	19.9 ± 2.5*** (15.6 – 25.5)	<0.0001
MCHC (g/dL)	32.5 ± 1.6(14.0 – 38.3)	32.2 ± 1.2* (29.8 – 35.8)	31.8 ± 3.0(25.3 – 41.8)	0.0015
RDW-CV (%)	16.4 ± 4.2(10.6 – 47.7)	20.3 ± 4.4*** (12.0 – 37.1)	23.6 ± 4.3*** (15.0 – 34.0)	<0.0001
CE-electropherogram	EA	EA	EFA	
Hb E + A_2_ (%)	25.2 ± 4.4(8.4 – 33.4)	57.6 ± 7.1(40.5 – 69.4)	52.5 ± 8.3(38.7 – 63.1)	<0.0001
Hb A_2_ (%)	3.4 ± 0.5(1.8 – 7.7)	7.0 ± 1.5(4.5 – 10.6)	6.7 ± 1.3(4.3 – 11.5)	<0.0001
Hb F (%)	0.4 ± 1.3 (0.0 – 10.1)	3.9 ± 3.1(0.0 – 9.7)	20.7 ± 10.5(10.5 – 40.7)	<0.0001

The significant differences (*p* value) were tested by the Kruskal-Wallis test.

* = *p* < 0.05 compared to heterozygous HbE.

** = *p* < 0.01 compared to heterozygous HbE.

*** = *p* < 0.0001 compared to heterozygous HbE.

**Figure 4.**
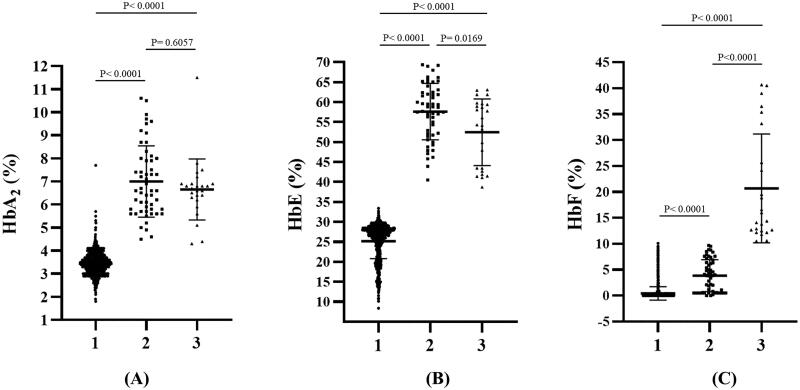
Comparison of HbA_2_ levels (A), HbE levels (B), and HbF levels (C) quantified by the capillary electrophoresis system among patients with heterozygous HbE (1), HbE-β^+^-thalassemia with low HbF expression (2), and HbE-β^+^-thalassemia with high HbF expression (3). Hb: hemoglobin.

**Figure 5.**
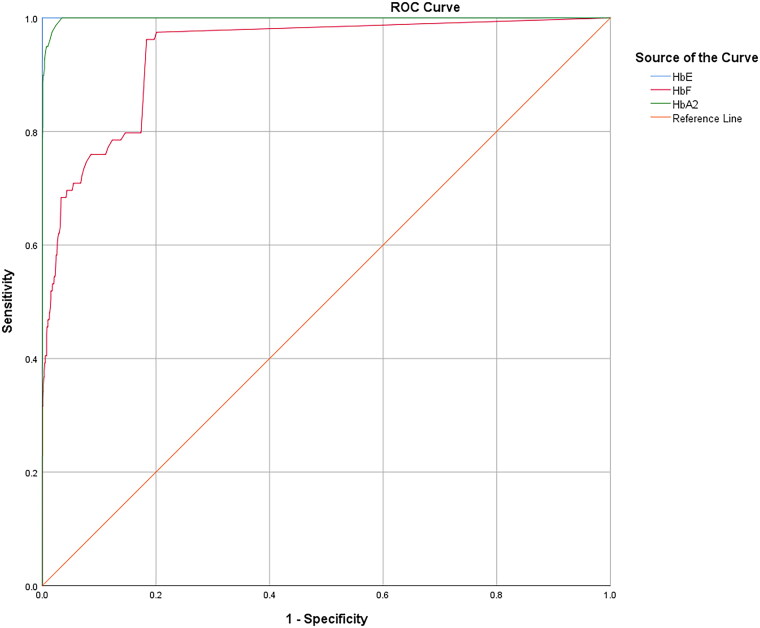
ROC analyses of the HbE, HbF, and HbA_2_ levels for differentiating patients with HbE-β^+^-thalassemia from HbE heterozygotes.

The distributions of HbA_2_, HbE, and HbF levels in patients with HbE heterozygosity and HbE-β^+^-thalassemia (with low- and high-HbF levels) are shown in Figure 4. The percentage of HbA_2_ observed among patients with HbE heterozygosity, low-HbF HbE-β^+^-thalassemia, and high-HbF HbE-β^+^-thalassemia was 3.4 ± 0.5, 7.0 ± 1.5, and 6.7 ± 1.3, respectively. The percentage of HbE in patients with HbE heterozygosity, low-HbF HbE-β^+^-thalassemia, and high-HbF HbE-β^+^-thalassemia was 25.2 ± 4.4, 57.6 ± 7.1, and 52.5 ± 8.3, respectively. The percentage of HbA_2_ and HbE in patients with HbE-β^+^-thalassemia, with both low and high HbF levels, was significantly higher than that in patients with HbE heterozygotes (*p* < 0.0001). HbA_2_ levels exhibited no statistically significant differences between HbE-β^+^-thalassemia patients with low and high HbF levels (*p* = 0.6057) (Figure 4A). However, the HbE levels of patients with HbE-β^+^-thalassemia with low HbF levels were significantly higher than those of patients with high HbF levels (*p* = 0.0169) (Figure 4B). Additionally, the percentage of HbF in patients with HbE-β^+^-thalassemia with low and high HbF levels was significantly higher than that in patients with HbE heterozygotes (Figure 4C). We observed that, although the HbF level was significantly different between patients with HbE heterozygosity and HbE-β^+^-thalassemia with low HbF levels, there was some overlap in HbF levels among these patients. The proportion of HbE-β^+^-thalassemia patients disguised with low HbF levels was 2.6% (54/2108). ROC curves were analyzed to investigate the cut-off values of HbA_2_, HbE, and HbF for ascertaining HbE-β^+^-thalassemia (Figure 5 and Table 2). The AUC for HbF level was 0.937 (95% confidence interval (CI):0.911–0.963), and the cutoff value was found to be 0.05, with a sensitivity of 97.5%, specificity of 20.2%, and Youden’s index of 17.7%. For the HbA_2_ level, the AUC was 0.998 (95% CI:0.997–1.000) and the cut-off value was found to be 4.25, with a sensitivity of 100.0%, specificity of 3.5%, and Youden’s index of 5.1%. Furthermore, the HbE level was found to be an excellent parameter with an AUC of 1.000 (95% CI:1.000–1.000), and the cut-off point was between 33.05 and 36.05, with 100.0% sensitivity, specificity, and Youden’s index. To accommodate the variation in Hb analysis results among different analyzers, we proposed a cut-off HbE level of 35.0%. Therefore, participants with an HbE level < 35.0% were likely to be HbE heterozygotes, whereas those with HbE levels ≥35.0% were suspected of having HbE-β^+^-thalassemia, requiring further mutation analysis.

**Table 2.** Sensitivity, specificity, false positive rate (FP), false negative rate (FN), and youden’s index based on varying cutoffs of HbE, HbF, and HbA_2_ values.

Parameters tested	Sensitivity	Specificity	FP	FN	Youden’s index (%)
Hb E (%)	AUC = 1.000	95% CI = 1.000 – 1.000			
>32.45	100	99.9	0.1	0	99.9
>33.05	100	100	0	0	100
>36.05	100	100	0	0	100
>39.60	98.7	100	0	1.3	98.7
>40.80	97.5	100	0	2.5	97.5
>41.25	96.2	100	0	3.8	96.2
**Hb F (%)**	**AUC = 0.937**	**95% CI = 0.911 − 0.963**			
>0.05	97.5	20.2	79.8	2.5	17.7
>0.15	97.5	20.1	79.9	2.5	17.6
>0.25	96.2	19.7	80.3	3.8	15.9
>0.35	96.2	19.0	81.0	3.8	15.2
>0.45	96.2	18.4	81.6	3.8	14.6
**Hb A_2_ (%)**	**AUC = 0.998**	**95% CI = 0.997 − 1.000**			
>4.15	100	5.1	94.9	0	5.1
>4.25	100	3.5	96.5	0	5.1
>4.35	98.7	2.4	97.6	1.3	1.1
>4.45	97.5	1.7	98.3	2.5	−0.8
>4.55	96.2	1.4	98.6	3.8	−2.4

**Figure 6.**
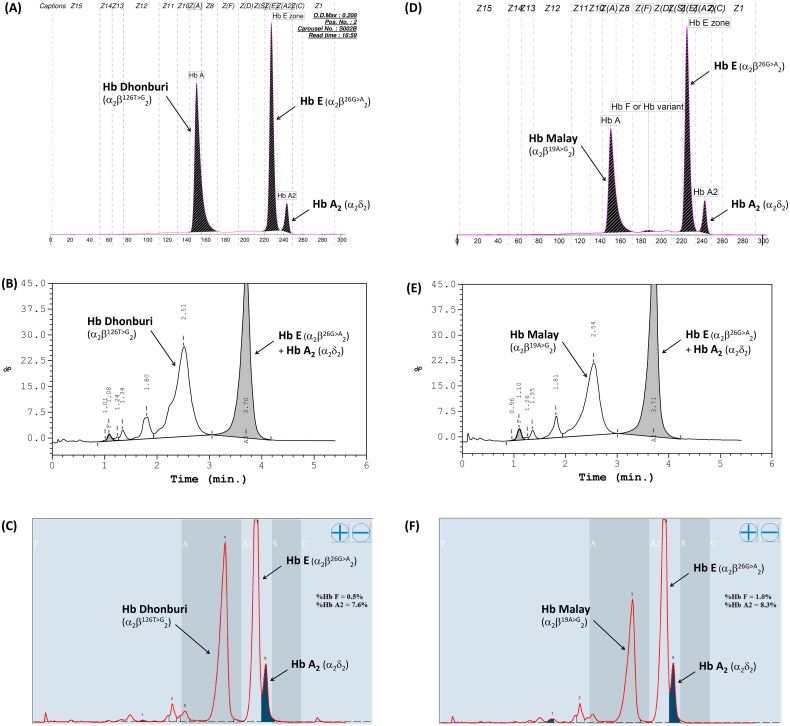
Hb analysis of Hb Malay (A-C) and Hb Dhonburi (D-F) using capillary electrophoresis (A&D), HPLC-VARIANT II (B&E), and HPLC-Premier resolution (C&F).

Among the 79 patients with HbE-β^+^-thalassemia, various β-thalassemia mutations were identified in patients with low HbF levels compared with those with high HbF levels. The most common β^+^-thalassemia mutation was the nt-28(HBB:c.-78A > G) mutation, which was observed in 59.3 and 92.0% of the patients with low and high HbF levels, respectively. Other mutations with different frequencies and hematological parameters are presented in Table 3. The G to A transition in codon 19 and the T to G transition in codon 126 of the β-globin gene, corresponding to Hb Malay (HBB:c.59A > G) and Hb Dhonburi (HBB:c.380T > G), respectively, were identified in only seven patients with low HbF levels. These mutations produced a β-globin variant with the same migration time as HbA in the electropherogram (Figure 6A and B). The electropherogram was likely to be an HbE heterozygote owing to a fraction of HbE, and another Hb that seemed to be assigned as HbA was distinctly observed. Similarly, Hb analysis using HPLC methods (VARIANT II, Premier resolution) revealed a high peak of HbE (retention time (RT) 3.70–3.71 min for VARIANT II, and 5.45–5.47 min for Premier resolution) and a high-value peak of another Hb was eluted at RT 2.51–2.54 min (VARIANT II) and 4.66–4.69 min (Premier resolution), which was the position of HbA. This Hb pattern was observed in both variants (Figure 6).

**Table 3.** β-globin mutations, frequency, hematological data, HbA_2_, HbE, and HbF levels in participants having HbE-β^+^-thalassemia with low- and high-HbF expression. Values are presented as means ± standard deviations or as raw data where appropriate.

Groups	HbE-β^+^/thalassemia with low-HbF level (*n* = 54)					HbE-β^+^/thalassemia with high-HbF level (*n* = 25)	
	β^-28(A>G)^	β^-31(A>G)^	β^-87(C>A)^	β^19(A>G)^ or Hb Malay	β^126(T>G)^ or Hb Dhonburi	β^-28(A>G)^	β^-87(C>A)^
β-globin mutations	(HBB:c.-78A > G)	(HBB:c.-81A > G)	(HBB:c.-137C > A)	(HBB:c.59A > G)	(HBB:c.380T > G)	(HBB:c.-78A > G)	(HBB:c.-137C > A)
Number	32	13	2	4	3	23	2
Frequency (%)	59.3	24.1	3.7	7.4	5.5	92.0	8.0
RBC (×10^12^/L)	5.11 ± 1.0	5.80 ± 1.8	6.11, 4.58	5.41 ± 0.4	4.7 ± 0.6	4.69 ± 0.9	3.31, 4.73
Hb (g/dL)	9.3 ± 2.0	11.5 ± 4.4	10.6, 8.1	10.1 ± 1.1	8 ± 0.2	9.2 ± 1.5	8.1, 9.7
Hct (%)	29.0 ± 5.9	35.5 ± 14.2	30.7, 25.0	31.2 ± 4.3	26.0 ± 1.5	29.0 ± 4.6	23.8, 29.3
MCV (fL)	56.0 ± 5.0	61.3 ± 10.2	50.2, 54.5	57.4 ± 4.3	55.4 ± 3.4	63.0 ± 7.8	59.3, 62.0
MCH (pg)	18.0 ± 1.4	19.8 ± 3.3	17.3, 17.7	18.7 ± 0.8	17.0 ± 1.6	19.9 ± 2.5	24.7, 20.4
MCHC (g/dL)	32.2 ± 1.2	32.2 ± 1.4	34.5, 32.4	32.7 ± 1.2	30.7 ± 0.9	31.8 ± 3.0	41.8, 33.0
RDW-CV (%)	21.6 ± 4.7	17.9 ± 3.1	20.0, 18.1	18.4 ± 1.5	18.7 ± 5.0	23.6 ± 4.3	15.0, 20.7
Hb A_2_ (%)	7.4 ± 1.5	6.8 ± 1.6	4.5, 4.6	6.1 ± 0.9	6.1 ± 1.2	6.7 ± 1.3	6.2, 6.3
Hb E + A_2_ (%)	61.1 ± 5.0	49.2 ± 3.1	58.7, 57.5	58.7 ± 2.0	50.2 ± 9.2	52.5 ± 8.3	57.8, 54.2
Hb F (%)	3.9 ± 3.3	3.9 ± 2.0	8.5, 9.5	3.2 ± 2.9	0.3 ± 0.3	20.7 ± 10.5	12.2, 16.0

Hb: hemoglobin.

**Figure 7.**
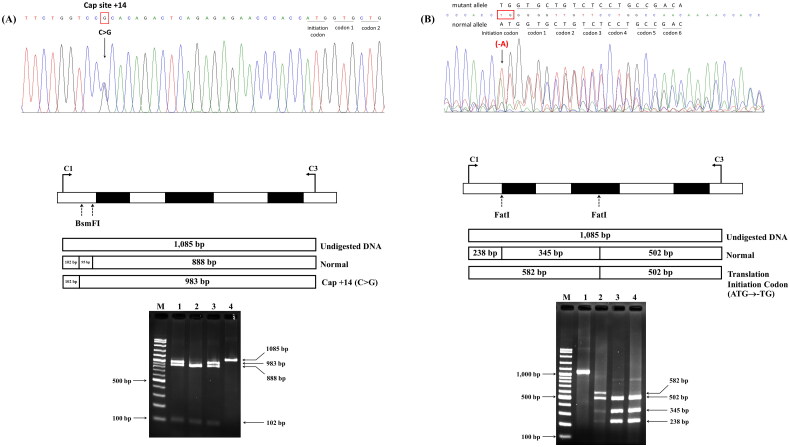
Identification and Confirmation of the Cap +14(C>G) mutation and initiation codon (ATG>-TG) of the α_2_-globin gene by nucleotide sequencing and PCR-RFLP.

DNA analysis of the α-globin gene showed α-thalassemia mutations in 46.3% (25/54) of patients with HbE-β^+^-thalassemia with low HbF levels and 8.0% (2/25) of those with high HbF levels. HbCS had the highest proportion of common α-thalassemia mutations found in the former groups. In addition, we also identified the compound heterozygous HbCS with 3.7 kb deletional α^+^-thalassemia and α-thalassemia 1 (SEA), the latter expressed the HbH-CS phenotype. Remarkably, no HbCS peaks were observed in the CE electropherogram of all patients with HbCS. An unexpected mutation, located in the promoter region of the α_2_-globin gene, Cap +14(C>G) [HBA2:c.-24C>G], was identified in two patients with HbE-β^+^-thalassemia with low HbF levels, whose β-globin genotype was β^E^/β^Malay^ and β^E^/β^Dhonburi^. This α-globin mutation has not previously been described in the Thai population. Additionally, a deletional A at the translation initiation codon of the α_2_-globin gene (ATG>-TG) [HBA2:c.1delA] was identified in one patient with HbE-β^+^-thalassemia with a low HbF level, whose β-globin genotype was β^E^/β^−28^. These mutations were confirmed by PCR-RFLP using *Bsm*FI and *Fat*I. As shownin Figure 7, digestion of the 1085-bp fragment with *Bsm*FI gave rise to three different fragments and the 983-bp fragment in the mutated allele, indicating the presence of the [HBA2:c.-24C>G (or HBA1)] mutation. Digestion with *Fat*I resulted in four fragments; the 582-bp fragment indicating the presence of a mutation in the translation codon. The hematological parameters, HbE, HbA_2_, and HbF, of each corresponding HbE and α-globin genotype are summarized in Table 4. Remarkably, patients with HbE-β^+^-thalassemia with low and high HbF levels who had normal α-globin genes showed no differences in hematological parameters, HbE, and HbA_2_ levels.

**Table 4.** α-globin genotypes, hematological parameters, HbE, HbA_2_, and HbF levels among HbE-β^+^-thalassemia with low- and high-HbF levels subjects. Values are presented as means ± standard deviations or as raw data where applicable.

Genotypes	RBC	Hb	Hct	MCV	MCH	MCHC	RDW-CV	Hb E + A_2_	Hb A_2_	Hb F
	(10^12^/L)	(g/dL)	(%)	(fL)	(pg)	(g/dL)	(%)	(%)	(%)	(%)
HbE-β^+^-thal with low Hb F (*n* = 54)										
β^E^/β^+^, αα/αα (29)	5.29 ± 1.4*	9.9 ± 3.2*	31.0 ± 10.5*	58.4 ± 7.6*	18.7 ± 2.4*	32.1 ± 1.1*	21.3 ± 5.4*	55.3 ± 6.6*	6.8 ± 1.3*	3.5 ± 3.0
β^E^/β^+^, α^-24C>G^α/αα (2)	4.11, 5.41	9.4, 10.5	27.1, 33.6	61.9, 62.1	19.5, 19.4	31.5, 31.3	19.7, 20.6	40.5, 57.0	7.4, 4.9	0.5, 6.9
β^E^/β^+^, α^TIC(A-G)^α/αα (1)	2.91	5.4	16.6	56.9	18.5	32.5	19.2	60.0	5.6	8.9
β^E^/β^+^, -α^3.7^/αα (3)	5.0 ± 0.6	10.3 ± 2.6	32.7 ± 8.3	57.0 ± 7.0	17.8 ± 2.3	31.4 ± 1.5	21.1 ± 1.1	58.9 ± 7.1	8.1 ± 2.1	5.3 ± 4.6
β^E^/β^+^, -α^4.2^/αα (2)	5.9, 5.6	10.8, 10.8	33.8, 33.7	57.6, 59.9	18.4, 19.6	31.9, 32.7	17.0, 18.0	64.9, 51.8	8.0, 6.6	7.6, 2.8
β^E^/β^+^, α^CS^α/αα (9)	5.65 ± 1.0	10.1 ± 1.6	30.8 ± 4.4	54.9 ± 5.4	17.9 ± 1.2	32.7 ± 1.4	19.3 ± 1.8	61.2 ± 4.7	7.4 ± 2.1	4.2 ± 2.9
β^E^/β^+^, -α^3.7^/α^CS^α (1)	5.9	11.1	35.1	59.3	18.8	31.7	19.9	59.4	NA	8.8
β^E^/β^+^, --^SEA^/αα (6)	4.68 ± 0.8	9.3 ± 2.2	27.9 ± 6.2	55.2 ± 4.1	18.4 ± 1.3	33.3 ± 1.6	17.5 ± 2.2	62.7 ± 1.7	8.5 ± 1.7	3.2 ± 2.8
β^E^/β^+^, --^SEA^/α^CS^α (1)	5.60	7.7	26.0	45.0	14.0	30.0	21.0	56.2	7.5	7.2
HbE-β^+^-thal with High Hb F (*n* = 25)										
β^E^/β^+^, αα/αα (23)	4.62 ± 0.8	9.2 ± 1.4	29.0 ± 4.0	63.0 ± 7.9	20.2 ± 2.4	32.4 ± 2.5	23.9 ± 4.4	51.7 ± 8.2	6.4 ± 0.9	21.4 ± 10.7
β^E^/β^+^, --^SEA^/αα (1)	7.06	11.0	41.1	58.2	15.6	26.8	21.9	61.9	11.5	12.5
β^E^/β^+^, -α^3.7^/αα (1)	3.98	7.1	28.0	71.0	18.0	25.3	19.6	61.9	7.5	12.7

* No statistically significant difference (*p*-value >0.05) as compared to HbE-β^+^-thalassemia with high-HbF levels and normal α-globin gene.

The distribution of the genotypes and alleles of the −158 (C-T) ^G^γ *Xmn*I polymorphism (SNP rs7482144) among the 79 patients with HbE-β^+^-thalassemia with low and high HbF levels is summarized in Table 5. The 54 patients with low HbF levels showed a relatively high proportion of the CT/TT genotype (66.7%), whereas the allelic C frequency was relatively high (63.9%). Among the 24 patients with high HbF levels, the CT/TT genotype was identified as relatively high (approximately 80.0%), and the C allele was identified in approximately 60% of patients. Comparison of the proportion of genotypes and alleles of the examined ^G^γ-*Xmn*I polymorphism between the low and high HbF level groups revealed no statistically significant difference (*p* > 0.05).

**Table 5.** The genotypes and allele proportion of -158 (C-T) ^G^γ *Xmn*I polymorphism among patients with HbE-β^+^-thalassemia with low- and high-HbF expression.

Gene	SNPs ID	Genotype/Allele	Low Hb F expression (*N* = 54)	High Hb F expression (*N* = 25)	*P-*value
^G^γ-*Xmn*I	rs7482144 (C–T)	CC genotype	18 (33.3%)	5 (20.0%)	0.292
		CT + TT genotype	36 (66.7%)	20 (80.0%)	
		C allele	69 (63.9%)	30 (60.0%)	0.724
		T allele	39 (36.1%)	20 (40.0%)	

## Discussion

HbE (beta26(B8)Glu > Lys;HBB:c.79G > A) is a β-thalassemic Hb variant whose frequency throughout Thailand is relatively high, together with a high prevalence of β-thalassemia [4]. Thus, the coexistence of HbE with β-thalassemia leading to HbE-β-thalassemia disease is common in this country. The clinical and hematological severity of the disease are variable. The type of β-thalassemia mutation and DNA polymorphisms within the β-globin gene cluster determine the clinical severity [8,26,27]. The characteristic phenotype of HbE-β^+^-thalassemia usually presents as mild-to-moderate anemia (Hb levels ranging from 7.0–9.0 g/dL), markedly low MCV and MCH, and elevated HbF expression (>10% of total Hb) with Hb analysis exhibiting an EFA pattern [2,26]. However, patients presented with major peaks of HbE and HbA on Hb analysis, quite similar to that of the HbE heterozygote, but the level of HbE was relatively high compared to subjects who were pure HbE heterozygotes. Furthermore, non-elevated HbF levels are regularly encountered in routine laboratories for thalassemia diagnosis. Based on the Hb analysis results, these patients were misdiagnosed as HbE heterozygotes or those with abnormal Hb, which had motility behavior similar to that of HbA. Therefore, the patient did not receive appropriate genetic counseling, treatment, or disease management.

In this study, 79 (3.7%) out of 2133 patients had varying HbF levels and β^+^-thalassemia mutations in *trans* to the β^E^-globin allele. Of these, 54 had relatively low HbF levels, whose Hb type was read as HbE and HbA (Figure 1C), while the remaining 25 patients had relatively high HbF levels, with HbE, HbA, and HbF (Figure 1D). This demonstrated that approximately 2.6% (54/2108) of the patients in our cohort could be misdiagnosed as HbE heterozygotes unless DNA analysis was performed. The hematological parameters of HbE-β^+^-thalassemia with both low and high HbF levels showed a more severe thalassemia syndrome phenotype, with average Hb concentrations <10 g/dL, markedly decreased MCV and MCH, and distinctly increased RDW-CV compared to HbE heterozygotes (Table 1). Nevertheless, differentiation of HbE-β^+^-thalassemia from HbE heterozygosity using hematologic parameters alone is challenging because we found that some patients with HbE-β^+^-thalassemia were not anemic or slightly anemic and had slightly abnormal red cell parameters, similar to those observed in HbE heterozygotes [7,28]. Remarkably, a markedly elevated HbE level of 38.7–69.4% was observed in patients who had HbE-β^+^-thalassemia, while HbE levels of approximately 8.4–33.4% were observed in HbE heterozygotes (Figure 4). This is similar to the results of previous studies [2,7,11]. Additionally, relatively high HbA_2_ levels, separated and calculated by CE, were observed in patients with HbE-β^+^-thalassemia (Figure 4). Thus, Hb levels may be useful markers for differentiating HbE-β^+^-thalassemia from HbE heterozygotes. The ROC curves constructed to calculate the AUC for HbA_2_, HbE, and HbF revealed an excellent AUC (1.000) for HbE (Figure 5, Table 2). An accurate cut-off of ≥ 35.0% for HbE levels was proposed to differentiate patients with HbE-β^+^-thalassemia from HbE heterozygotes, which exhibited 100.0% sensitivity, specificity, and Youden’s index. Although this differentiation was not evaluated in independent prospective cases, our results showed that HbE level may be a robust indicator for differentiating patients with HbE-β^+^-thalassemia from HbE heterozygotes, which is simple, less time-consuming, and inexpensive to evaluate. This HbE level could be applied in clinical settings to screen cases for the possibility of HbE-β^+^-thalassemia requiring definite diagnosis by DNA analysis, leading to a significant reduction in the misdiagnosis of HbE heterozygosity and resulting in DNA analysis oversight.

The types of β-thalassemia mutations identified in the 79 patients with β^+^-thalassemia were similar to the β-thalassemia mutations most commonly identified previously in Thai β-thalassemia carriers and patients with HbE-β^+^-thalassemia [15,29]. Remarkably, two β-globin chain variants (Hb Malay and Hb Dhonburi) were identified in only seven patients with low HbF levels presenting the thalassemia intermedia phenotype (Table 3). This finding is concordant with previous reports [2,30–32]. Hb Malay is caused by a mutation in codon 19 (AAC > AGC) (Figure 2E) and usually presents with a thalassemia intermedia phenotype when co-inherited with HbE and has more severe anemia when associated with β^0^-thalassemia [2,30,31].

Hb Dhonburi or Hb Neapolis are caused by a GTG to GGG mutation at codon 126 associated with a mild β-thalassemia phenotype [32], which is rarely reported in the Thai population. A previous study on the α/β globin mRNA ratio revealed that the average ratio observed in Hb Dhonburi carriers was relatively high (1.7) compared with that of the normal group (1.1), confirming that this variant is a β^+^-thalassemia [2]. In our study, the three patients with coinheritance of HbE and Hb Dhonburi were presented with an intermediate phenotype with an RBC count of 4.7 ± 0.6 × 10^12^/L, Hb level of 8.0 ± 0.2 g/dL, and Hct of 26.0 ± 1.5. This result was similar to that observed in another Thai patient who coinherited HbE and Hb Dhonburi [33]. Hb separation in Hb Malay and Hb Dhonburi using CE and HPLC (VARIANT II) was also studied. The results showed that they could not identify aberrant Hb representing Hb Malay and Hb Dhonburi in either HbE heterozygotes or HbE compound heterozygotes [33]. However, we could separate these variants using the new HPLC (Premier resolution with high-resolution mode), which, to the best of our knowledge, has not been previously used and revealed that Hb Dhonburi eluted at the same RT as HbA (Figure 6). Our results emphasize that these two variants are difficult to distinguish from HbA using Hb electrophoresis and HPLC because β^Malay^ and β^Dhonburi^ globin chains have biochemical properties similar to those of HbA [32].

In our study, variable amounts of HbF were detected in addition to HbE in patients with HbE-β^+^-thalassemia. However, similar β^+^-thal mutations were identified in 54 and 25 patients with markedly different HbF expression levels, respectively. Therefore, the β-thal mutation alone does not account for the observed wide expression of HbF, and other modifying genetic factors may be responsible. Previous studies have demonstrated that many genetic factors are associated with HbF expression in HbE-related disorders [8–10,26,34]. The αβ-thalassemia mutation has also been explained as one of the factors contributing to HbF expression. As expected, we found a relatively high proportion of αβ-thalassemia mutations (approximately 46.3%) among the 54 patients with low HbF levels, whereas it was only 8.0% among the 24 patients with high HbF levels. It is noteworthy that the HbE level among patients co-inheriting the α-thalassemia mutation was consistently elevated, albeit with more pronounced defects in α-globin gene chain synthesis (Table 4). HbCS was the highest proportion with missing HbCS peak on the CE electropherogram. Interestingly, we found that two patients had complex thalassemia syndrome: one had co-inheritance of compound heterozygous HbCS with -α^3.7^, having genotype -α^3.7^/α^CS^α, β^E^/β^−28^, and the other had co-inheritance of compound heterozygous HbCS with α-thalassemia 1 (–^SEA^/α^CS^α, β^E^/β^−28^), diagnosed hematologically as CSEFABart’s disease, an uncommon form of thalassemia intermedia rarely observed in the Thai population. Defects in the three α-globin genes in this patient did not appear to affect HbE expression because of the consistently elevated HbE observed (Table 4). Interestingly, HbCS was not seen on the CE electropherogram from either patient, and additional HbBart’s (0.4%) were observed in the latter. Therefore, there is a possibility of misdiagnosis in these patients unless a complete DNA analysis is performed. Unexpectedly, we found that the Cap +14(C>G) mutation located in the promoter region of the α_2_-globin gene might be responsible for α^+^-thalassemia because of the partially abolished translation identified in two patients, one with the β^E^/β^Malay^ genotype and the other with the β^E^/β^Dhonburi^ genotype. The Cap +14(C>G) mutation has been found in patients with Hb J-Meerut and Hb Winnipeg [α75(EF4)Asp→Tyr (α2); HBA2: c.226G > T (or HBA1)] [35,36], but this mutation has not yet been observed in the Thai population. To the best of our knowledge, this is the first report of doubly coinherited HbE with Hb Malay and Hb Dhonburi with the Cap +14(C>G) mutation causing the β-thalassemia intermedia phenotype in Thai patients. Additionally, we found that the ATG to -TG mutation in the initiation codon of the α_2_-globin gene completely abolished translation in the β^E^/β^−28^ patient without an apparent effect on HbE expression. This mutation causes α^+^-thalassemia, which has been reported in Thai patients with HbH [37]. The interactions between β-thalassemia and different α-thalassemia mutations identified in patients with HbE-β^+^-thalassemia in our study led to more than nine genotypes (Table 4), which cause complex thalassemia syndromes, making accurate diagnosis with routine laboratory testing, using hematological parameters alone, difficult.

The markedly different proportion of α-thalassemia observed between patients with low and high HbF levels supports the hypothesis that α-thalassemia mutations have a favorable influence on HbF expression in patients with HbE-β^+^-thalassemia in our study. The coinheritance of α-thalassemia has been reported to be strongly associated with amelioration of clinical severity in HbE-β-thalassemia syndrome [8,26,38–40]. Therefore, the patients in this study with low HbF levels, presenting with hematological features similar to those with high HbF levels, may have co-inherited α-thalassemia (Tables 1 and 3). Concomitant inheritance of α-thalassemia should lead to a more balanced β-globin and α-globin chain synthesis and should be responsible for decreased reactivation of the γ-globin gene in the disease. However, co-inheritance of α the-thalassemia mutation was not observed in 29 (53.7%) patients with low HbF levels, possibly because of other inherent factors associated with low HbF expression in those patients. Additionally, an investigation of the −158 (C-T) ^G^γ *Xmn*I polymorphism revealed no significant difference in the proportion of the genotype and allele between patients with low and high HbF levels (Table 5). In our study, this polymorphism was less associated with HbF levels in patients with HbE-β^+^-thalassemia. However, we found a high proportion of the C allele (63.9% and 60.0%) among patients with low and high HbF levels, respectively, which was similar to that observed by Winichagoon et al. [39]. The C allele is associated with β^+^-thalassemia. Our results emphasized that the C allele was associated with the type of βmutation rather than with HbF expression in patients with HbE-β^+^-thalassemia. Previous studies have demonstrated that in HbE-β-thalassemia, the T allele or T/T genotype is also associated with increased HbF and milder anemia [6,41]. However, in our study, the T allele was identified in a relatively low proportion of patients with both low and high HbF levels (Table 5). Overall, our results indicate that α-thalassemia mutations favorably influenced HbF levels in patients with HbE-β^+^-thalassemia and increased susceptibility to low HbF levels. However, additional unknown modulating factors in patients require further investigation.

## Conclusion

HbE-β^+^-thalassemia has a widely variable expression of HbF, and many patients whose Hb type is read as HbE and/or HbA can be misdiagnosed with HbE-β^+^-thalassemia. Therefore, molecular analysis of the β-globin gene should be performed to improve the accuracy of diagnosis. We also found that α-thalassemia mutations are a common factor favorably decreasing HbF expression, resulting in complex thalassemia syndromes requiring complete DNA analysis; therefore, the presence of α-thalassemia mutations should always be assessed to provide complete genetic information. Furthermore, an HbE cutoff value of ≥35.0% may be a potential indicator for effectively differentiating HbE-β^+^-thalassemia from HbE heterozygosity. Patients whose Hb analysis showed HbE and HbA and elevated HbE, regardless of the amount of HbF, could not be concluded to have HbE heterozygosity unless DNA analysis was performed. This study provides details regarding HbE-β^+^-thalassemia that may help in diagnosis, especially in areas where HbE and β-thalassemia are both prevalent.

### Consent to publish

Consent to publish patient details was obtained from all individuals included in the study.

## Data Availability

Data supporting the findings of this study are available from the corresponding author upon reasonable request.
